# Highs and Lows in Calicivirus Reverse Genetics

**DOI:** 10.3390/v16060866

**Published:** 2024-05-28

**Authors:** Ángel L. Álvarez, Aroa Arboleya, Fábio A. Abade dos Santos, Alberto García-Manso, Inés Nicieza, Kevin P. Dalton, Francisco Parra, José M. Martín-Alonso

**Affiliations:** 1 Instituto Universitario de Biotecnología de Asturias (IUBA), Departamento de Bioquímica y Biología Molecular, Universidad de Oviedo, 33006 Oviedo, Spain; 2 Instituto Nacional de Investigação Agrária e Veterinária, 2780-157 Oeiras, Portugal

**Keywords:** calicivirus, reverse genetics, infectious clone, RNA virus

## Abstract

In virology, the term reverse genetics refers to a set of methodologies in which changes are introduced into the viral genome and their effects on the generation of infectious viral progeny and their phenotypic features are assessed. Reverse genetics emerged thanks to advances in recombinant DNA technology, which made the isolation, cloning, and modification of genes through mutagenesis possible. Most virus reverse genetics studies depend on our capacity to rescue an infectious wild-type virus progeny from cell cultures transfected with an “infectious clone”. This infectious clone generally consists of a circular DNA plasmid containing a functional copy of the full-length viral genome, under the control of an appropriate polymerase promoter. For most DNA viruses, reverse genetics systems are very straightforward since DNA virus genomes are relatively easy to handle and modify and are also (with few notable exceptions) infectious per se. This is not true for RNA viruses, whose genomes need to be reverse-transcribed into cDNA before any modification can be performed. Establishing reverse genetics systems for members of the *Caliciviridae* has proven exceptionally challenging due to the low number of members of this family that propagate in cell culture. Despite the early successful rescue of calicivirus from a genome-length cDNA more than two decades ago, reverse genetics methods are not routine procedures that can be easily extrapolated to other members of the family. Reports of calicivirus reverse genetics systems have been few and far between. In this review, we discuss the main pitfalls, failures, and delays behind the generation of several successful calicivirus infectious clones.

## 1. Introduction

Unlike classical genetics, in which efforts are made to discern an unknown genotype from the observation of the phenotype, reverse genetics relies on the direct alteration of the genotype and the study of the effects of such alterations on the phenotype. In the molecular virology field, the term reverse genetics refers to a set of methodologies in which changes are introduced into the viral genome and their effects on the generation of infectious viral progeny and their phenotypic features are assessed in terms of viability, gene essentiality, virulence, attenuation, tropism, host range, resistance to antivirals, etc.

Reverse genetics emerged thanks to recombinant DNA technology, which made possible the isolation and cloning of genes and their modification through mutagenesis. Most viral reverse genetics studies depend on the capability of rescuing an infectious wild-type viral progeny from cell cultures transfected with an “infectious clone”. An infectious clone generally consists of a circular DNA plasmid containing a functional copy of the full-length viral genome, under the control of an appropriate polymerase promoter. For most DNA viruses, reverse genetics systems are straightforward since DNA virus genomes are relatively easy to handle and modify and are also (with few notable exceptions) infectious per se. This is not true for RNA viruses, whose genomes always need to be reverse transcribed into cDNA before any modification—such as restriction enzyme digestion, gene knock-out or knock-in, site-directed mutagenesis, ligation, etc.—can be performed, as this molecular toolbox is not fully available for RNA alteration [1].

Even though fully functional infectious clones from ribosome-ready positive-sense RNA viruses are theoretically simpler to obtain compared to those for negative-sense or double-stranded RNA genomes, reverse genetics systems for *Caliciviridae* are exceptionally challenging. In part, this may be due to the small number of members of this family that propagate in cell cultures. Despite success in the rescue of *Vesivirus felis* (formerly, *Feline calicivirus*, FCV) from cDNA clones more than 20 years ago [2], reverse genetics methods are not routine procedures that can be quickly implemented for other caliciviruses. Obtaining the RNA transcript from a cDNA clone of the viral genome is a prerequisite for any reverse genetics system but, once in the cytosol, the ability of such transcripts to trigger virus replication and recovery is not guaranteed, which is why reports of calicivirus reverse genetics systems have been few and far between [1,3,4].

The methodology used for infectious clone rescue seeks to reproduce the effects of viral infection, by transfecting permissive cells, either with a cDNA vector (generally a plasmid) containing the viral genome under the control of an appropriate promoter or with synthetic RNA produced through in vitro transcription of the former. Regardless of the strategy followed, obtaining an infectious clone provides a powerful tool for the use of reverse genetics techniques through the manipulation of the viral genome and the study of the effects of certain gene changes (point mutations, deletions, insertions, inversions, or translocations) on the biology of viruses, their replication cycle, the role of viral proteins in pathogenicity, or in the interactions between viruses and the immune response components [3,5,6].

Additionally, reverse genetics opens new avenues for vaccine development based on the possibility of controlled virus attenuation and the use of replicons or defective viruses as vectors for the expression of proteins with potential biotechnological applications. Replicons are RNAs derived from viral genomes that retain the ability to replicate autonomously in the cytoplasm. Usually, these replicons harbor partial or complete deletion of the genes encoding the structural proteins to prevent the formation of infectious particles. Such deletions also allow the replicon system to accept the insertion of foreign genes of interest without exceeding its coding capacity, which could compromise RNA replication. Sometimes, the supplementation of virion structural proteins in trans allows the packaging of the replicon within viral particles. These de novo-produced viral particles are defective in their ability to produce progeny since their genomes lack the sequence for structural proteins but can be engineered to express foreign genes of interest for a single round of infection. Except for retroviruses, replicons derived from positive-sense RNA viruses do not integrate exogenous genetic information into the host cell genome. Many systems have been described for heterologous gene expression based on infectious cDNA clones of filoviruses [7], influenza viruses [8] (reviewed in [9]), reoviruses [10,11,12], rotaviruses [13,14] (reviewed in [15]), bornaviruses [16], bunyaviruses [17], picornaviruses [18], flaviviruses [19], alphaviruses [20], and coronaviruses [21,22,23,24], among others.

## 2. The *Caliciviridae*: Genome Organization, Gene Expression, and Replication Strategies

**Figure 1:**
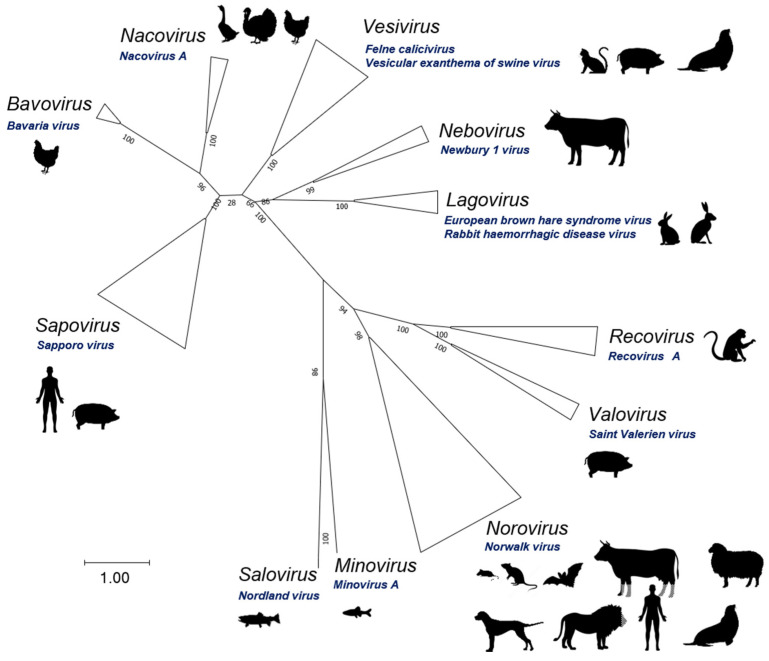
Phylogenetic tree showing the 11 established calicivirus genera: *Lagovirus*, *Recovirus*, *Valovirus*, *Norovirus*, *Minovirus*, *Salovirus*, *Sapovirus*, *Bavovirus*, *Nacovirus*, *Vesivirus,* and *Nebovirus*, in accordance with the 2023 release of the International Committee on Taxonomy of Viruses (ICTV) report. These genera group 13 recognized species in total. The tree was adapted from [26] and is based on the amino acid sequences of the major structural protein (VP1). Bootstrap support shown is based on 500 replicates. This figure does not make a distinction among genogroups and genotypes included in some of the genera (such as *Norovirus*). For details, visit the ICTV’s *Caliciviridae* website (https://ictv.global/report/chapter/caliciviridae/caliciviridae, accessed on 20 March 2024). The silhouettes refer to the hosts of the isolates included in the phylogenetic tree but are not intended to represent all potential hosts.

The family *Caliciviridae* comprises viruses that infect vertebrates, such as birds, reptiles, and mammals, including humans (reviewed in [25]). In recent years, the number of genera making up the family increased from 5 to 11, including a total of 13 recognized species [4,26], which are shown in Figure 1.

Caliciviruses have a linear, single-stranded, positive-sense RNA genome of 7.3 to 8.5 kb in length. The genome is flanked by well-conserved untranslated regions (UTRs) at the 5′ and 3′ termini [27]. The 5′–UTR (ranging from 4 to 19 nucleotides) always starts with the 5′–pGpU–3′ sequence [28], and the most 5′–G is covalently linked to a nucleotidylated tyrosine residue of a small basic virus-encoded polypeptide, namely “viral protein genome-linked” (VPg) [29]. A polycistronic coding region is followed by a downstream 3′–UTR spanning 46 to 108 nucleotides and a poly-A tail of variable length [28].

A subgenomic RNA (sgRNA), that is the 3′ co-terminal with the genomic RNA (gRNA), is produced during replication and packaged into progeny virions along with the gRNA. The sgRNA has a similar organization in all genera, being equivalent to the last third of its corresponding genome, and is also VPg-linked at the 5′ terminus [30].

**Figure 2:**
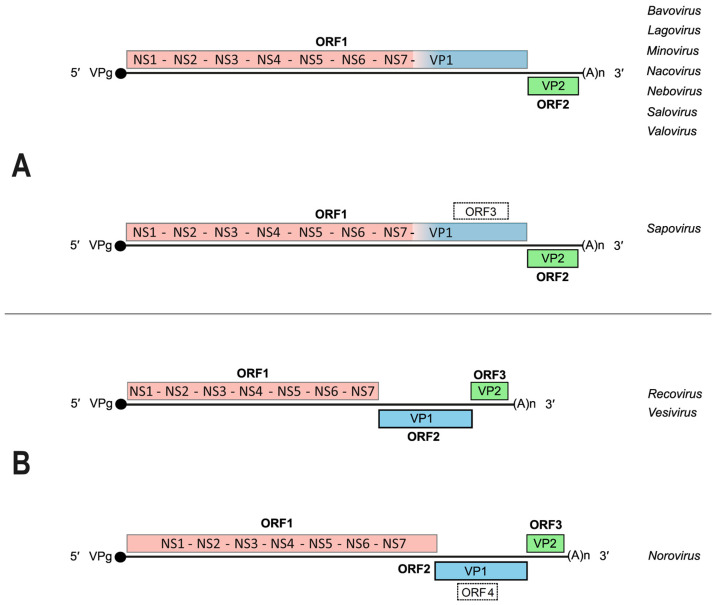
Two general models for calicivirus genome organization: the “two-ORF” model (**A**) and the “three-ORF” model (**B**). Open reading frames (ORFs) are indicated as well as the location of regions coding for known enzymatic activities and viral functions. NS1–NS7: non-structural proteins 1–7; VP1: viral protein 1 (major capsid protein); VP2: viral protein 2 (minor capsid protein). Adapted from [26,37]. See further details in the text.

The genome organization has established itself as a distinctive feature of each genus within the *Caliciviridae* (Figure 2). Two clearly different models exist based on the number of open reading frames (ORFs) present in the coding region of the genome. Some calicivirus genomes are made up of two main ORFs (genera *Bavo*–, *Lago*–, *Mino*–, *Naco*–, *Nebo*–, *Salo*–, *Valo*–, and *Sapovirus*) (Figure 2A), while the other comprise three (genera *Reco*–, *Vesi*–, and *Norovirus*) (Figure 2B). However, an additional nested ORF has been found within the major capsid protein (VP1) coding sequence in *Sapovirus* and *Norovirus* genomes (represented in Figure 2 with dashed lines). These cryptic ORFs have been designated as ORF3 and ORF4, respectively [26], and their putative functions will be briefly mentioned elsewhere in this review.

For all *Caliciviridae* members, ORF1 encodes a large precursor polyprotein that is co- and post-translationally cleaved into precursors and mature polypeptides by the *cis*-acting protease NS6 that is part of such polyprotein. The extent of proteolytic processing and therefore the mature products display marked differences between calicivirus genera [31,32]. During the translation of viral RNA, the 5’most nonstructural gene products (NS1–NS7) appear first, followed by the VP1 and the minor capsid protein (VP2) [3].

In the “two-ORF” model of genome organization, VP1 is the most C-terminal portion of the ORF1-encoded polyprotein. Therefore, theoretically, VP1 could be released from the polyprotein upon NS6-mediated proteolytic cleavage following translation [33]. However, VP1 is mainly produced from the sgRNA during the late stages of infection, when higher amounts of the capsid protein are required for packaging [34]. For the viruses organized according to the “three-ORF” model, the ORF1-encoded polyprotein ends with NS7 and VP1 is only produced during sgRNA translation. In both models, the ORF closest to the 3′–end is the smallest and contains information for the synthesis of VP2. This ORF generally overlaps to some extent with the previous ORF [35,36].

The roles of all non-structural proteins have not yet been fully elucidated; however, it is clear that NS3 possesses NTPase/helicase activity; NS5 (also referred to as viral protein genome-linked, VPg) is a paradigm-breaking polypeptide since it acts as a protein primer for RNA synthesis initiation [38]. VPg also serves in the recruitment of translation initiation factors (such as eIF4A, eIF4E, and eIF4G) onto ribosomes during viral RNA translation [39,40,41,42], a function which, when absent, is reportedly recoverable by simply adding a regular eukaryotic 7-methylguanosine cap structure to the 5′ end of viral RNA [2,43]. NS6 is a protease very similar to the picornavirus 3C cysteine protease, which is why the former is referred to as 3C-like protease in the literature. Calicivirus 3C-like cysteine proteases are considered members of a family of chymotrypsin-like serin proteases that contain a cysteine instead of serine as the nucleophile in the active site. NS6 cleaves viral polyproteins and precursor proteins and contributes to the assembly of viral RNA replication factories [31,33]. NS7 is an RNA-dependent RNA polymerase (RdRp), also known as a viral replicase (reviewed in [25]). NS1/2 and NS4 have not been fully characterized but it has been hypothesized that, as they contain membrane-spanning hydrophobic domains, they might be involved in the rearrangement of cell organelle membranes during the assembly of membrane-associated virus replication factories [44]. It has also been suggested that they act as virulence factors (e.g., viroporins and antiviral immune response suppressors), thus playing roles in viral pathogenicity and epidemiological fitness (for a comprehensive review of calicivirus non-structural proteins and their analogs in picornaviruses, refer to Smertina et al. [45]).

Although the taxonomy of caliciviruses is mainly based on sequence comparisons, there are some genome peculiarities within some of the genera regarding gene expression and processing of precursor proteins. For example, the ORF2 of all members of the *Vesivirus* genus produces a precursor VP1 protein that is further cleaved by the viral protease, removing a small N-terminal peptide and yielding the mature major capsid protein [31,46]. The small peptide released upon VP1 processing was named ‘leader of the capsid protein’ (LC) and is reportedly required for viral replication [47]. In FCV, the LC is considered essential for infection in vitro and to produce the characteristic virus-induced cytopathic effect (CPE) in host cells [48], including the appearance of cell refringence and rounding, detachment of the infected cell monolayer from the culture vessel, and ultimately, cell lysis and death.

Additional genus-specific extraordinary features come from the members of the *Vesivirus*, whose proteolytic and RNA polymerase enzymatic activities are exerted by a unique bi-functional polypeptide (referred to as NS6–7^Pro-Pol^), as no further proteolytic cleavage has been reported in their NS6 and NS7 junctions. Differences also exist in the processing of the NS1/2 junctions, with the *Naco*–, *Reco*–, and *Valovirus* genera members showing no processing, *Vesi*–, *Lago*–, *Nebo*–, and *Sapovirus* NS1/2 being processed by the viral NS6, and *Norovirus* members’ NS1/2 junctions processed by the caspase–3 cellular protease (reviewed in [45]).

As previously stated, an additional ORF has been identified in sapoviruses (ORF3) and murine norovirus (MNV) (ORF4), which appears nested within the sequence coding for VP1. While the functions of sapovirus ORF3 remain elusive [49], MNV’s ORF4 encodes a protein named virulence factor 1 (VF1), with a potential role in regulating the innate immune response and apoptosis during the infection [32,50].

## 3. Calicivirus Replication Cycle

Following adsorption and entry into a susceptible and permissive cell, the viral genome is released in the cytoplasm and the translation of the ORF1 starts [3]. The VPg protein is crucial in this step as it functions as a proteinaceous cap substitute for translation factors and ribosome positioning, in contrast to the eukaryotic mRNAs that require a 5′–cap structure for eIF4F cap-binding complex recruiting and protein synthesis initiation. The first product synthesized is the large polyprotein encoded by ORF1, which includes NS1–5, the cis-acting cysteine protease NS6 for its own processing, and the viral replicase NS7.

Following translation, the genomic RNA (starting from its 3′–end) serves as a template for NS7-driven de novo RNA synthesis, yielding a transient double-strand RNA (dsRNA) intermediate [3,51]. The newly synthesized negative-sense RNA strand then serves as a template for the VPg-primed synthesis of multiple copies of both gRNA and sgRNA that, in turn, is used as the substrate for several rounds of translation, allowing the accumulation of all viral proteins, especially the structural proteins VP1 and VP2 [3,30]. The VP2-encoding ORF is the second ORF within the sgRNA, just downstream of the VP1-encoding ORF. Because the eukaryotic translation machinery only reads and translates the first ORF, most eukaryotic mRNAs are monocistronic, and the translation of VP2 in the context of bicistronic caliciviral sgRNA is challenging. The mechanism underlying VP2 synthesis has not been fully elucidated in all caliciviruses, but several studies have suggested a termination/reinitiation mechanism where the ribosome backtracks from the first ORF’s stop codon to the next ORF’s initiating AUG. Apparently, this phenomenon strictly depends on the presence of the above-mentioned terminal codon and a specific signal sequence called the ‘termination upstream ribosome binding site’ (TURBS), which encompasses approximately 80 nucleotides upstream from the stop codon [35,36,52,53].

**Figure 3:**
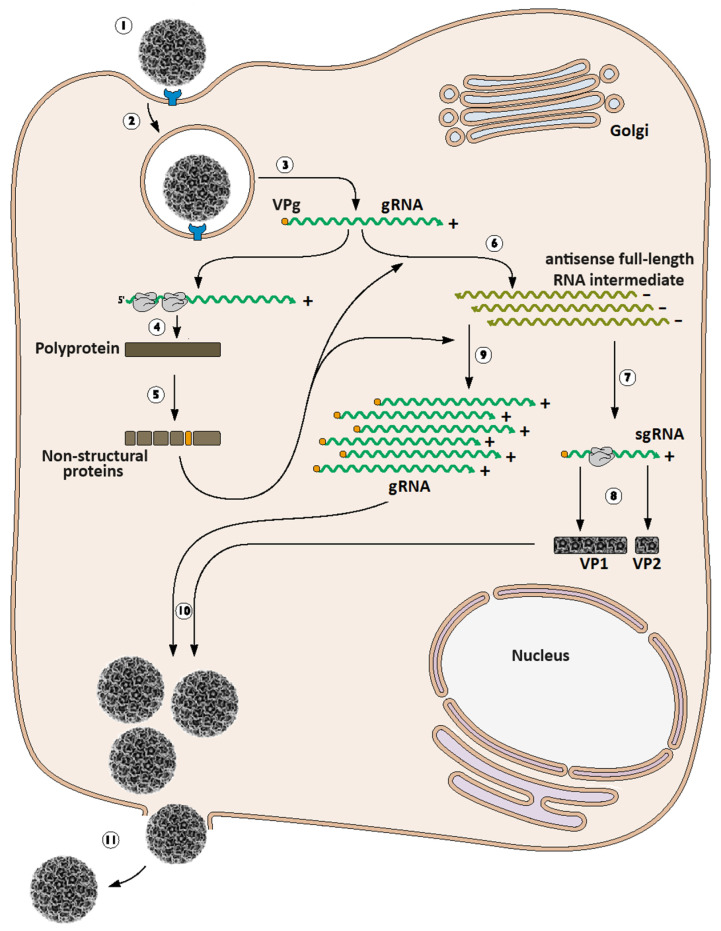
Schematic representation of the replication cycle of a hypothetical *Caliciviridae* member. Calicivirus major replication steps include virus attachment or adsorption (1), endocytosis-mediated internalization or entry (2), translocation and uncoating of viral gRNA into the cytoplasm (3), gRNA translation leading to ORF1-encoded polyprotein synthesis (4) and further autoproteolytic processing that yields the non-structural mature polypeptides (5), synthesis of the genome-length antisense RNA intermediate (6) that serves as the template for both the sgRNA synthesis (7) that is subsequently translated into structural proteins (VP1 and VP2) (8) and the generation of multiple copies of the gRNA (9). The newly synthesized viral components (e.g., capsid proteins, gRNA, and sgRNA) are put together into the progeny viral particles (10), which are ultimately released from the infected cell (11) during late events, concomitantly associated with cell lysis and death. Inspired by and adapted from [63]. See further details in the text.

When critical levels of structural proteins and gRNA are reached, capsid assembly, genome packaging, and release of progeny virions take place, ultimately leading to cell lysis. As for most viruses with isometric (e.g., icosahedral) symmetry, capsid self-assembly is thermodynamically favored and spontaneously occurs under specific conditions [54]. The role of VP2 in this process has been reported [55]. It is noteworthy that the VPg-linked sgRNA is packaged together with the VPg-linked gRNA inside progeny virions, although the biological significance of this phenomenon remains unknown [30]. A schematic representation of the replication cycle of a hypothetical calicivirus can be seen in Figure 3. Further details of the replication cycle of a model calicivirus are comprehensively reviewed in [3,56].

## 4. In Vitro Study of Caliciviruses

### 4.1. The Challenges of Reverse Genetics

A permissive and productive cell culture system is a major step towards the functional analysis of viral proteins and opens up the possibility of efficiently recovering (rescuing) viruses, a pivotal prerequisite for reverse genetics studies [3]. A few caliciviruses are cultivable in vitro. Some notable examples include MNV, which replicates well in murine macrophages, RAW264, BV–2, and WEHI cells, etc. [57,58]; FCV, in Crandell–Rees feline kidney (CRFK) cells [59]; Recovirus A, in the monkey kidney (LLC-MK2) cell line [60]; and rabbit vesivirus in Vero cells [43,61]. However, for many members of the family, a robust and reproducible cell-culture system has not been reported yet, and this has hampered the study of the calicivirus replication cycle and has delayed the development of reverse genetics systems [3]. This is especially true for human noroviruses, whose propagation in BJAB cells—a B lymphocyte-derived cell line—has been described, although the virus yield was rather poor. [62].

Calicivirus genomes are infectious per se and are immediately translated by the eukaryotic ribosomes, following entry into the host cell. This means that, if introduced into permissive cells, full-length viral RNA should ultimately lead to productive infection (virus rescue or recovery). Although the term ‘virus rescue’ has systematically been employed as a synonym of reverse genetics, the latter more properly refers to the attempts to recover virions from mutated or genetically altered viral genomes, provided that the rescue of wild-type virions from unaltered genomes has previously been achieved in a reproducible manner so that it could be set up as a parallel control assay in every reverse genetics experiment.

As previously mentioned, the advent of recombinant DNA technology provided a vast toolbox for DNA alteration (such as hundreds of different restriction endonucleases, DNA modifying enzymes, ligases, etc.). However, RNA modification is not as straightforward as DNA engineering. In the case of RNA viruses, a complete genome-length cDNA clone, which is obtained through reverse transcription (RT), must be assembled into an appropriate expression vector before any genetic alteration (reverse genetics study) can be performed. While advances in RT technology mean that the synthesis of a full-length cDNA is relatively simple, the choice of a sequence after cloning may not be so: due to the lack of proofreading activity of most RdRps, RNA virus stocks are often a mix of slightly different (polymorphic) genomes, referred to as virus quasispecies. The first challenge encountered during the setup of an RNA virus reverse genetics system is the fact that a single cDNA clone only represents a unique genome sequence within the RNA quasispecies. Because the cloning procedure does not assess sequence quality in terms of virus fitness, a selected sequence may harbor lethal point mutations that render it replication-incompetent [64,65]. Such defective RNA molecules would most likely be naturally removed from the pool in subsequent rounds of replication; nevertheless, they may end up being the chosen cDNA picked from bacterial colonies during cloning [1].

Depending on the viral genome size, the construction of a genome-length cDNA vector supporting the synthesis of infectious transcripts can be long and tedious [6,66,67], but once a reproducible workflow is established from the genotype (viral genome) to the phenotype (rescued virions), it represents a major leap for virus research. An established and reproducible infectious clone provides a powerful tool for reverse genetics experiments, in which researchers directly manipulate the viral genome (for example, introducing point mutations, deletions, insertions, inversions, or translocations) and further assess the effects of such manipulations on the phenotype in terms of fitness, replication competence (virus yield and attenuation), tropism, host range, virulence, pathogenicity, immunogenicity, etc. Also, reverse genetics finds applications in the study of viral protein functions and vaccine development [5].

For many viral genomes, another relevant challenge is the intrinsic instability of large full-length constructs and their toxicity for bacteria, which makes the preparative purification of genome-length cDNA-containing plasmids a very tricky task. Uncontrolled sequence rearrangements and mutations that render the derived RNA transcripts non-functional have been systematically reported [68,69,70]. Cloning the genomic cDNA of interest into an expression vector, trying a different bacterial strain, lowering the culture temperature (e.g., using 30–32 °C instead of 37 °C), or even increasing the bacterial culture volume to compensate for the reduced plasmid yield during maxi-preps could eventually help [43].

**Figure 4:**
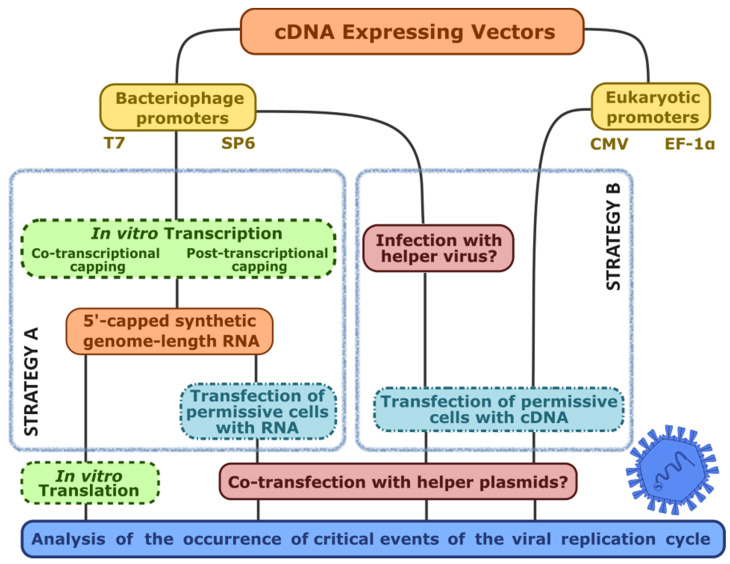
Flowchart depicting the main strategies for the establishment of a calicivirus reverse genetics system. Strategy A: Transfection with either co- or post-transcriptionally capped in vitro synthesized RNA. Depending on the selected promoter in the cDNA, genome-length RNA transcripts can be produced with any commercially available in vitro transcription system (such as those based on T7 or SP6 bacteriophages RNA polymerases). Strategy B: Transfection with cDNA-expressing vector, provided that the appropriate RNA polymerase for cDNA transcription will simultaneously be expressed (or supplemented in trans) within the transfected cells. In vitro translation of synthetic RNA can be attempted as a complementary assay to check translation-worthiness of transcripts. Attempts to revert systematic virus recovery failure can be made by co-transfecting the defective infectious clones with plasmids expressing each of the viral proteins. See further details in the text.

Figure 4 summarizes the two main strategies for the recovery of infectious virus from full genome-encoding cDNA. The permissive cell culture can be transfected with either synthetic genome-emulating, potentially infectious RNA obtained through in vitro transcription (Strategy A), or with the genome-length cDNA properly cloned into an expression vector under the control of an appropriate RNA polymerase promoter (Strategy B). The simultaneous occurrence of the genome-length cDNA-expressing vector and the relevant RNA polymerase will drive transcription. Regardless of the strategy used, the presence of infectious genome-length RNA will ultimately lead to productive infection: viral protein synthesis, viral genome replication, sgRNA synthesis, virus assembly, maturation, and virion progeny release (virus rescue).

When cloning a viral genome in the form of cDNA into an expression vector or plasmid, several prokaryotic or eukaryotic RNA polymerase promoters can be used to drive transcription. SP6, T3, and T7 bacteriophages RNA polymerase promoters are the most popular prokaryotic promoters and are usually used when in vitro transcription and further RNA transfection is the goal (Figure 4, Strategy A), although eukaryotic promoter-based RNA synthesis systems are also available (e.g., Cytomegalovirus (CMV) promoter-driven in vitro transcription kit).

The synthesis of cDNA is performed by RT using viral genomic RNA as a template and an oligonucleotide primer annealing within the 3′–end of the viral genome (usually oligo-dT since calicivirus RNAs are 3′–polyadenylated). Secondary structures are a distinctive feature of single-stranded RNA (ssRNA). When present in viral genomic RNA, highly stable variants of such secondary structures may pose a major obstacle to RT fidelity: sometimes the RT is unable to unwind these structures while copying and just skips them, leading to sequence gaps or other types of mutations that ultimately compromise the recovery of viable viruses [70]. After ribonuclease H-mediated removal of viral RNA, first-strand DNA is amplified by PCR using a second primer. In general, the larger the genome size, the lower the probability of obtaining an error-free genome-length cDNA copy in a row. Therefore, sometimes this goal is achieved by assembling several smaller PCR-generated cDNA pieces into the whole genomic cDNA by means of ligation following enzymatic cutting of the amplicons’ ends with unique restriction endonucleases [43,70] or through Gibson assembly, a molecular cloning technique that joins multiple DNA fragments in a predetermined order without the need for restriction enzyme sites. This technique relies on the assembly of overlapping fragments, typically generated by PCR, followed by their combination using three enzymes: a 5′–exonuclease, a DNA polymerase, and a DNA ligase, all within an isothermal reaction [71,72].

Overall, for a cDNA-derived RNA transcript to mimic the viral genome as closely as possible and trigger a productive infection, extreme care should be taken during the cDNA construct design, regarding the selection of the expression vector, the cloning strategy, the choice of promoter, the delivery method, etc. Not only do the coding regions of the genomic sequence need to be accurate but also both the 5′ and 3′ termini due to their crucial roles in translation and replication processes. It is generally accepted that the presence of non-viral nucleotides upstream from the 5′–end of RNA transcripts (potentially coming from the vector multiple cloning site or promoter) drastically reduces (or completely abolishes) infectivity, which jeopardizes virus recovery.

Regarding this approach, it is worth recalling that in vitro synthesized transcripts need to be “translation-ready”, a quality naturally conferred to calicivirus RNAs by the VPg protein. However, the in vitro covalent linkage of synthetic transcripts to VPg is technically challenging [41]; therefore, the generation of a capped 5′–end (as a VPg substitute) is required for the translation initiation of synthetic RNAs. A synthetic cap structure has been used (m7G[5′]ppp[5′]G) at the 5′–end of in vitro-transcribed RNA allowing the recovery of infectious viruses [2], albeit sometimes with low efficiency [73]. The 5′–cap structure can be either co-transcriptionally incorporated into nascent RNA or post-transcriptionally added to RNA. Alternatively, the introduction of an internal ribosome entry site (IRES) within the 5′–UTR of RNA transcripts bypasses the requirement for a covalently linked VPg or a 5′–cap structure. In vitro translation of synthetic RNA can be attempted as a complementary assay to evaluate the translation-worthiness of transcripts [74].

For the transfection of vectors containing transcription-ready cDNA (Figure 4, Strategy B), eukaryotic promoters, such as the CMV, SV40, or EF-1α promoters, are often used. The synthesis of genome-emulating RNA transcripts takes place in the nucleus and is catalyzed by the eukaryotic cell RNA polymerase II. If a eukaryotic promoter cannot be used or a prokaryotic promoter is preferred, the transcription of viral genome-like cDNA can alternatively be controlled by a prokaryotic promoter such as that of T7 RNA polymerase, as long as this phage’s transcriptase, naturally absent in eukaryotic cells, is supplied in trans. This supplementation can be achieved by infecting the cells with a ‘helper virus’ or by using a recombinant cell line expressing the T7 phage RNA polymerase. The helper virus is usually a recombinant poxvirus expressing the T7 RNA polymerase, such as vaccinia virus-T7 (rVV-T7), Ankara-modified vaccinia-T7 (rMVA-T7), or fowlpox virus-T7 (rFPV-T7). The infection with a helper virus is generally performed prior to cDNA transfection.

One advantage of the use of helper poxviruses in calicivirus reverse genetics includes the high levels of expression of the heterologous RNA polymerase, which, in turn, guarantees a high transcription rate for calicivirus cDNA, and the fact that poxviruses encode their own RNA capping enzymatic complex to make caliciviral transcripts ready for translation. In addition, the complete poxvirus replication cycle occurs in the cytoplasm, thus avoiding potential deleterious effects due to the interaction of calicivirus RNA transcripts with the nucleus (e.g., unwanted splicing or other RNA editing processes). The major disadvantages associated with helper virus usage during calicivirus reverse genetics are their toxicity for the host cells, the difficulty in distinguishing between the helper virus-associated CPEs and those CPEs potentially attributable to calicivirus rescue, and the need for specific methods for helper virus removal in case of successful calicivirus recovery. In this regard, the fowlpox virus is preferred over the vaccinia virus because the former displays an abortive replication cycle in mammalian cells, which prevents a fowlpox virus progeny from being formed, thus no interference with calicivirus rescue occurs (reviewed in [75]).

The effectiveness of a specific helper virus in the recovery of a particular calicivirus is not guaranteed. Instead, the degree of success achieved varies between different virus systems and often results from multiple trial-and-error experiments. For example, the rescue of Cowden I virus (aka., porcine enteric calicivirus, PEC) (species *Sapovirus sapporoense*; ICTV 2023 release) from a cDNA clone systematically failed when using rMVA-T7 as a helper virus because of the strong CPE recorded in the host cell line [76], while rMVA-T7 was apparently useful to achieve some degree for human norovirus replication in 293T [77]. rMVA-T7 did not allow murine norovirus recovery upon transfection of RAW264.7 cells; however, the virus rescue was successful when the helper virus rFPV-T7 was used instead [73].

### 4.2. The Hallmarks of a Promising Virus-Expressing cDNA

**Figure 5:**
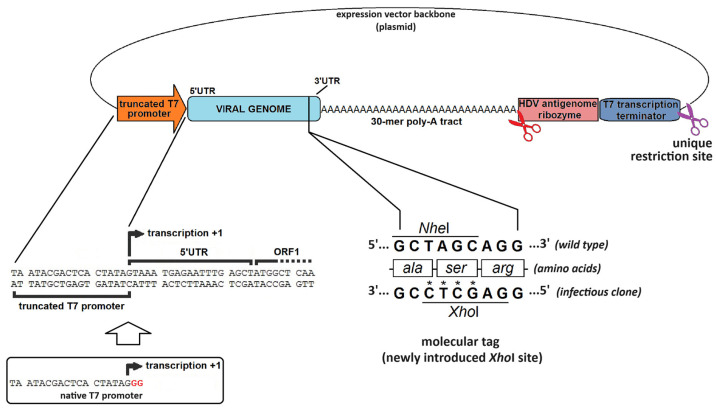
Schematic representation of a hypothetical calicivirus infectious clone, showing the main genetic regulatory elements that should be included in an ideal construct to generate functional “genome-emulating” RNA transcripts with a defined length, a precise 5′ terminus, and a sufficiently long polyadenylated free 3′–end. Asterisks (*) represent the nucleotide substitutions introduced to generate the molecular tag. See further details in the text (Adapted from [43]).

Figure 5 shows a schematic diagram of the design of a functional calicivirus T7 promoter-based infectious clone, emphasizing aspects of the regulatory elements surrounding the viral genomic sequence. As previously mentioned, the T7 phage RNA polymerase promoter is widely preferred as it can tolerate a slight 2-nt truncation of its 3′–end without a significant decrease in RNA synthesis. This truncation is necessary to prevent the addition of these two non-viral nucleotides to the 5′–end of the transcript. Hence, the first nucleotide of the virus genome coincides with the transcription starting site. Similarly, there is evidence that a single point mutation in the 3′–UTR can thwart virus rescue in a reverse genetics system. Chaudhry et al. found that a single nucleotide change at the last residue of murine norovirus–1 (MNV–1) 3′–UTR was sufficient for its inactivation [73].

It is highly desirable for viral genome-emulating RNA to possess a long poly-A tail downstream from the 3′–UTR, as described for naturally occurring calicivirus genomes. The poly-A tail is an important determinant of RNA stability: it positively contributes to the RNA half-life and prevents 3′–exoribonucleases from reaching the coding region before replication is completed, thus supporting virus recovery. Interestingly, the poly-A tail has also been found to be essential for positive-sense RNA virus translation initiation, as the genome circularizes in a non-covalent fashion through protein–protein interactions that take place between poly-A interacting proteins and the translation initiation factors bound to the 5′–UTR (reviewed in [28]). Many successful reverse genetics systems included a poly-A tail in their design that is at least 30 nucleotides long [32,43,76].

The strategy followed to generate and deliver RNA transcripts must ensure these transcripts are of a defined length. For instance, if the cDNA vector is intended for in vitro transcription using a T7 phage RNA polymerase, a T7 terminator sequence at the end of the construct can be used to stop transcription at the correct location. If a termination sequence is not available for the RNA polymerase used, then a unique restriction site can be added downstream of the sequence to be transcribed. This allows the production of a linear cDNA of a defined length as the transcription template. The RNA polymerase will fall off the linearized template, terminating the polymerization, a technique often referred to as run-off. Some successful infectious clone designs combine both strategies: a cDNA-expressing vector is first linearized with the aid of a unique restriction enzyme, but the RNA polymerase is not expected to reach that region because transcription is supposed to stop at the upstream terminator (Figure 5) [43]. However, if in vitro transcription and further RNA delivery are not an option, a cDNA vector will be transfected instead, and it is usually delivered in its circular form, thus transcription termination mostly relies on terminator sequences. An accurate polyadenylated free 3′–end can also be generated by including an autocatalytic ribozyme sequence immediately downstream from the poly-A tract, such as the hepatitis delta virus (HDV) ribozyme. This sequence will fold and adopt a specific secondary structure capable of *cis*-acting autocatalytic cleavage, producing a break in the phosphodiester bond upstream from its most 5′ nucleotide, which ultimately separates it from the preceding poly-A tail without any additional nucleotides being included in the transcripts (Figure 5) [43,73,77,78].

Finally, the introduction of a molecular tag within the viral genome sequence is strongly recommended as it allows the cDNA-derived recovered virus to be unambiguously distinguished from the casuistic contamination of the reverse genetics experiment with a wild-type virus. This molecular tag should consist of an innocuous and easy-to-track short-sequence alteration, such as the introduction or removal of a notable restriction site that can be recognized in cDNA copies of the rescued viral sequence. Figure 5 (bottom right) shows an ingenious molecular tag within a calicivirus reverse genetics system that yields a virus progeny harboring a newly introduced *Xho*I restriction site (which is absent in the wild-type virus) and lacking an *Nhe*I site (which is present in the wild-type virus). This tag consisted of four single-nucleotide changes within the VP2-encoding region (ORF3), engineered in a way that the amino acid sequence of VP2 remained unchanged [43].

### 4.3. When Things Go Wrong: Interrogating the Viral Genome for the Occurrence of Replication-Critical Events

Despite the success in the rescue of many viruses from cDNA clones, reverse genetics methods are not routine procedures for all positive-polarity RNA viruses. The synthetic RNA must be recognized by the cellular machinery to produce viral proteins and subsequently interact with them appropriately to complete the viral replication cycle. There is also a need for the existence of permissive cell lines that should desirably be easily transfectable as well. The guidelines compiled in this section, based on the close examination of the calicivirus replication cycle, intend to bring a closer look at the events where virus rescue is more likely to fail and provide a practical approach for their troubleshooting.

For any reverse genetics system to succeed in generating infectious viral particles, every single event of the viral replication cycle should be achieved in a timely and efficient manner. In this regard, the assessment of the proper performance of viral cDNA constructs can be conducted through tailored assays designed to monitor the occurrence of such individual events directly or indirectly. Table 1 summarizes the critical steps in calicivirus replication that should be controlled during the replication cycle to rescue infectious virions using different reverse genetics strategies and distinct polymerase promoters.

After transfection with synthetic transcripts or cDNA-expressing vectors (Figure 4), the initial analytical approach entailed the microscopic examination of transfected cell monolayers, referred to as ‘passage 0’, to identify any CPE indicative of calicivirus replication as a result of successful virus recovery. In general, it must be considered that most of the transfection reagents available may cause a cytotoxic effect to some extent and that such an effect could sometimes be morphologically indistinguishable from virus-induced CPE observed in positive controls. Reportedly, the use of helper viruses, such as rFPV-T7 or rMVA-T7, to deliver RNA polymerase in trans may further increase the chance of observing misleading CPE, easily confounded with inexistent calicivirus recovery [43,73,74,79,80].

The difficulty in establishing the occurrence of viral rescue at passage 0 based solely on optical microscopy highlights the need for evaluating the infectivity of the supernatants from these cells (passage 0 supernatant) by inoculating them into new monolayers of susceptible and permissive cells. These inoculated cell cultures (passage 1) neither receive the toxic effect of the transfection reagent, which is diluted more than 10-fold in fresh culture medium, nor are infected with the helper virus as supernatants from passage 0 can be filtered before their subsequent inoculation into permissive cells. These helper poxviruses are usually larger than 0.2 μm in diameter [81] and therefore are mostly retained in the filters. Furthermore, in the case of FPV-T7, the only viral particles that could be present in passage 0 supernatants are those from the initial inoculum because this avian virus displays an abortive replication in mammalian cells and does not produce viral progeny [82], which also contributes to the fact that passage 1 cultures are not usually infected.

If no signs of CPE can be seen in cell cultures at passage 1, and there are no indications that the virus rescue has worked, the reasons could be related to the specific reverse genetics system used, the correctness of viral sequences, the quality of transfected nucleic acid, the cell line serving as the setting for passage 0, the promoters governing the transcription of the expression vectors, or the subcellular localization of RNA transcripts. In other words, when genome-length constructs fail to generate an infectious calicivirus progeny, efforts should be targeted at pinpointing the molecular factors responsible for their failure.

After cell transfections, the presence of intact full-length RNA in the cytosol is critical for subsequent events in the cycle to occur. A preliminary approach for troubleshooting will be the initial detection and analysis of the integrity of such RNAs, through RT-PCR (following plasmid template removal through extensive DNase treatment) and 5′–RACE assays, respectively. Even if RNA quantities in the cytosol were low, these assays are extremely sensitive and will most likely detect any RNA in the order of picograms. The low concentration of internalized synthetic RNA could be due to RNA degradation during the transfection process caused by the action of contaminating exogenous RNases or endogenous RNases activated as a cellular defense mechanism against the introduction of foreign RNA, especially if the capping procedure has not been efficient. Therefore, it is essential to control the quality and intactness of the present RNA molecules. 5′RACE (rapid amplification of cDNA 5′-ends) is a valuable technique for investigating the precise sequence of the RNA most 5′–end. The procedure involves utilizing PCR to amplify regions between the known segments of the sequence and non-specific tags attached to the ends of the cDNA [83].

RNAs that mimic the viral genome produced in the cytosol or introduced into it, either from the outside or from the cell nucleus, should be capable of translation, resulting in the viral polyprotein that contains the non-structural polypeptides. The detection of some of these polypeptides using WB analysis allows the demonstration, on one hand, that genomic RNAs present in the cytosol are functional for translation, and on the other hand, that the region with protease activity is also operative and could process the non-structural viral polyprotein. However, WB is limited not only by the availability of specific antibodies but also by the viral protein yield obtained during RNA translation. Thus, if such yield is not sufficient to be detected through WB, the use of a more sensitive detection technique should be applied, e.g., radioactive labeling followed by autoradiography.

If the replicative cycle proceeds normally, the translation of ORF1 should be followed by the synthesis of a negative-sense intermediate RNA (negative strand) catalyzed by the viral RdRp. The presence of these negative strands can be assayed using Northern blot. From an internal promoter on this negative strand, the viral RdRp synthesizes the sgRNA that gives rise to the structural proteins (VP1 and VP2). Additionally, from the 3′–end of the negative strand, the same enzyme produces multiple copies of the viral genome that will be packaged into newly produced viral capsids, forming the progeny.

The functionality of viral RdRp could indirectly be studied by means of the 5BR assay, which was initially developed for hepatitis C virus (HCV) [84] and subsequently adapted for calicivirus [85]. This assay utilizes components of one of the best-known signaling pathways involved in the innate antiviral response to infections with RNA viruses: the IFN-β synthesis pathway, activated by retinoic acid-inducible gene I (RIG-I) [85]. During viral RNA replication, RdRps transiently generate dsRNA intermediates, which are susceptible to being captured by the helicase domain of the RIG-I C-terminal region. Through the caspase activation and recruitment domain (CARD) in its N-terminal region, the activated RIG-I protein can interact with another CARD domain present in the mitochondrial antiviral-signaling protein (MAVS), a mitochondrial transmembrane protein. This interaction, in turn, activates a series of kinases (TBK1 and IKKε) that phosphorylate a cluster of serines in the C-terminal region of the transcription factor IRF3, promoting its dimerization and translocation to the nucleus. IRF3, together with NF-κB and ATF-2-c-Jun, forms a multiprotein complex or enhanceosome on the IFN-β promoter enhancer, triggering its transcription [86].

The 5BR assay artificially replicates this pathway using an expression vector based on the IFN-β promoter, in which the coding sequence for this cytokine has been replaced by the sequence of a luciferase. This vector is co-transfected simultaneously with a vector expressing the RIG-I protein, and the vector expressing the polymerase whose functionality is to be evaluated. The RNAs synthesized by transiently expressed RdRp can stimulate RIG-I-dependent reporter luciferase production via the beta interferon promoter, so the recorded luciferase activity is directly proportional to the amount of dsRNA and, therefore, to the RdRp activity [85].

## 5. Chronology of the Calicivirus Reverse Genetics

From the very first attempts of establishing a calicivirus reverse genetics system, the viral genome-length cDNA has been cloned into plasmid vectors in the context of several genetic regulatory elements, aiming at producing high-quality, genome-length, and potentially infectious RNA transcripts. These elements were briefly introduced in Section 4.2 and include the choice of an RNA polymerase promoter, a poly-A tract flanking the genome by its 3′ side, a transcription termination signal for the polymerase, whenever available, or a unique restriction site for endonuclease cut to make fixed-length transcripts via run-off. Additional elements for RNA 3′–end processing may optionally be added, which help to produce a consistent pool of RNA molecules with an accurate 3′–end, for example, the autocatalytic ribozyme.

**Figure 6:**
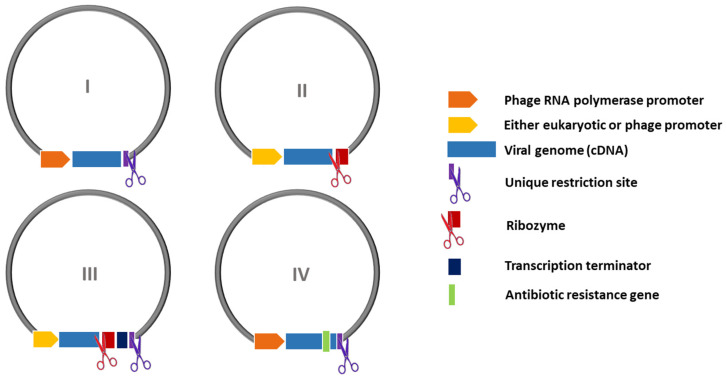
Schematic representation of four distinct arrangements (designs I–IV) of regulatory elements surrounding viral cDNA within expression vectors, observed among the reported calicivirus reverse genetics systems. Designs are numbered in increasing order of complexity. See further details in the text.

All these regulatory sequences have gradually been incorporated to improve the expression context of viral cDNA and the overall quality of RNA transcripts. Currently, the most reliable reverse genetics systems for caliciviruses combine several such improvements, arranged according to one out of four possible combinations (designs I–IV) that are schematically summarized in Figure 6. In addition, a brief review of the chronology of the different calicivirus reverse genetics systems is listed in Table 2.

The first infectious clone of a calicivirus was established for a cultivable strain (Urbana) of FCV [2]. The construction of a cDNA clone was achieved from a library by sequentially assembling three fragments into one genome-length copy. The design of the complete vector included the 5′ region of the viral genome juxtaposed to the promoter sequence of T7 bacteriophage RNA polymerase so that the transcription starting point of the T7 RNA polymerase matched the first nucleotide of the FCV genome. In the 3′ region, the sequence recognized by the restriction enzyme *Not*I was placed following the poly-A tail of the FCV cDNA sequence. This construction provides linear templates for in vitro synthesis of genome-length transcripts, but it involves the addition of two nucleotides of non-viral origin downstream of the poly-A tail. Transfection of these synthetic transcripts in CRFK cells resulted in an identical infectious process to that caused by RNA purified from virions. To confirm that the rescued virus came from the vector expressing the genome of cloned FCV, site-directed mutagenesis of the cDNA clone was performed to replace a *Stu*I site with a *Hind*III restriction site as a readily detectable molecular tag.

The synthetic RNA derived from this modified vector was also infectious and produced recombinant FCV virions, whose packaged genomic RNA contained the introduced mutation. The authors stressed the need for a cap analog added to the in vitro transcription since transcripts without a cap were not infectious. The FCV infectious clone was then used to investigate the proteolytic processing of capsid protein precursor [31] and the polyprotein encoded in ORF1 by introducing point mutations [87], as well as for determining the VPg residue responsible for binding to viral RNA [80]. These reverse genetics techniques have also been used to generate chimeric viruses in order to study antigenic variation in FCV [88] and to provide valuable information about virus replication mechanisms [47,48,89,90,91].

Following a similar approach, infectious clones were described for another FCV, vaccine strain 2024 [92], PEC [76], and Tulane virus (TV) [93]. In all cases, the expression vector contained the corresponding sequence of cDNA from the viral genome flanked by the T7 RNA polymerase promoter and by a unique restriction site to obtain linear templates for in vitro transcription. The incorporation of 5′–cap was essential for the functionality of synthetic RNA derived from these infectious clones, though their infectivity was approximately 100- to 1000-fold lower than those of RNA purified from virions [92]. For FCV and PEC, an alternative approach also allowed the rescue of infectious viruses: an rMVA-T7 was used to provide the polymerase in trans. Further transfection of a plasmid vector expressing the viral cDNA generated genome-length RNA transcripts in the cytoplasm. This strategy is thought to provide a higher number of RNA copies available to initiate viral replication [79,88,92].

This approach has also been used to study the replication and packaging of human noroviruses (species *Norwalk virus*, NV). The full-length viral cDNA from two different human norovirus isolates were independently produced using an identical approach, producing genome-length transcripts lacking nucleotides of non-viral origin [77,78]. Both cDNA clones were flanked by the T7 promoter (in their 5′–ends) and the sequence HDV ribozyme, followed by the T7 terminator signal at their 3′–ends. Although human noroviruses are difficult to propagate in vitro, the expression of these clones in cells infected with the vaccinia helper virus showed limited evidence of replication, such as the production of non-structural proteins and sgRNA. The rescue of viral particles was only possible when the system was supplemented with a plasmid expressing a cDNA copy of the viral sgRNA. In any case, such particles were not able to reproduce infection in new cell cultures, likely due to the lack of functional receptors (non-susceptible cells) [94]. The HDV ribozyme has proven effective in the rescue of other caliciviruses from cDNA, such as MNV-1 [95] and the FCV strain F4 [96], and in rescuing members of other viral families with different genome types, like influenza virus B (*Orthomyxoviridae*) [8] and rotaviruses (*Reoviridae*) [13]. Another fruitful reverse genetics strategy for human noroviruses employed the Gibson assembly method to generate an infectious cDNA clone from the complete genome of the HuNoV GII.4, Sydney subtype. This genome-length cDNA was inserted into a pcDNA3.1-based plasmid vector, downstream from the CMV promoter. The rescue of viral particles was accomplished after the transfection of Caco-2 cells in the absence of helper viruses [97].

Intracellular expression of a cDNA clone was also used to obtain an innovative reverse genetics system for MNV-1 [95]. The strategy chosen in this case was complex and involved the use of two baculoviruses. The first one contains the cDNA of the MNV-1 genome inserted between an inducible promoter for RNA polymerase II and the HDV ribozyme sequence. The second baculovirus expresses a transactivator of the inducible promoter, which allows transcription of the MNV-1 genome [95]. Thus, RNA transcription occurs in the nucleus and is post-transcriptionally processed (i.e., 5′–capped, ribozyme-mediated 3′–cut, etc.) and exported to the cytoplasm, where translation occurs for the synthesis of viral proteins and subsequent viral genomic replication. Although the exact position in which the polymerase begins transcription is not known, the virus recovered from the inducible baculovirus system showed the correct sequence at its 5′–end. This is important for the authenticity of the rescued virus since the manipulations carried out by reverse genetics should not include uncontrolled changes in the 5′–end, which could cause unknown effects. In this sense, strategies using promoters for eukaryotic polymerases can be less efficient when compared with systems using a bacteriophage promoter, which allows complete control over the first nucleotide in the RNA transcript.

Of the in vivo cDNA expression systems, the most common are those using the helper virus rMVA-T7 for two main reasons: (1) the T7 promoter ensures a controlled transcription initiation and (2) the cytoplasmic localization of the RNA transcripts produced by the rMVA-T7 RNA polymerase prevents possible modifications arising from exposure to the splicing and/or the nuclear-cytoplasmic transport machinery. As aforementioned, the rescue of FCV, PEC, and NV-1 from their respective cDNA clones with the help of the MVA-T7 virus has been possible [76,78,79]. However, a study by Chaudhry et al. [73] showed that the rMVA-T7 virus has negative effects on the replication of MNV-1. Conversely, they found that rFPV-T7 did not hamper the replication of RNA purified from MNV-1 virions. Moreover, they employed this helper virus to rescue MNV-1 using a vector where the MNV-1 cDNA clone is inserted between the T7 promoter and the HDV ribozyme sequence. These authors noted the importance of the sequence of RNA ends derived from a cDNA clone since the mutation of a single nucleotide preceding the poly-A tail has crucial effects on the functionality of the transcripts [73]. This infectious clone enabled a reverse genetics system useful for the study of the influence of viral RNA secondary structures in the replication of MNV [98,99] and to investigate factors that determine their virulence [100,101].

Initial studies with an MNV infectious clone indicated that, unlike the RNA purified from virions, transfection of synthetic transcripts (with or without a cap) did not result in the rescue of viral particles [73]. Nevertheless, in the studies that followed, the optimization of a reverse genetics system based on RNA transfection was described [32]. The system consists of post-transcriptionally capping of in vitro-synthesized transcripts with a recombinant guanylyl transferase from *Vaccinia virus*, which ensures efficiency close to 100% in the addition of a cap structure to the 5′–end of RNAs, much higher than the traditional procedure. Transfection of this RNA produced an infective process in cell culture, increasing the recovery of viral progeny in the order of 10 to 100 times in comparison to intracellular transcriptional systems aided by a helper virus [73] or a baculovirus system [95]. This reverse genetics system using optimized synthetic transcripts has allowed studies on the functional domains of various MNV genomic regions [102] and on other aspects of norovirus biology [51,103]. The persistence of the virus in infected animals for longer periods of time has been associated with secondary structures affecting the entire genome. The modification of these structures by reverse genetics has altered the persistence of the virus without modifying the kinetics of viral replication [101].

Some of these calicivirus infectious clones have allowed the expression of exogenous genes in eukaryotic cells. The *Aequorea coerulescens* green fluorescent protein (AcGFP) gene was inserted into the VP1 coding region of FCV RNA without affecting the ability of this rescued virus to replicate; the rescue of the replicon was possible by providing the capsid protein in trans. The resulting viral particles can infect a cell line susceptible to FCV infection, starting a new cycle of replication and expressing the fluorescent protein [92]. Later on, the FCV LC region was found to tolerate foreign insertions, such as AcGFP, *Discosoma* sp. red fluorescent protein (DsRed) [90], or mCherry [104], without hampering viral viability. In human norovirus, a GFP reporter construct containing the GFP gene in ORF1 produced complete virions that contain VPg-linked RNA, establishing a complete reverse genetics system expressed from cDNA with the EF-1α mammalian promoter and without the need for a helper virus [105]. Moreover, another fluorescent replicon was constructed by inserting the GFP between the NS3 (NTPase) and the NS4 (p22) genes through Gibson assembly, which produced a viral progeny that could be successfully detected and monitored in vitro using fluorescent microscopy [97].

Several replicons based on TV have been described, disclosing viral regions encoding structural proteins that are dispensable for RNA replication. A chimeric replicon, in which the TV VP1 gene has been replaced by the equivalent sequence of the NV VP1 gene, has also been obtained. This replicon causes CPE in transfected cells, but despite expressing the capsid protein, NV heterologous capsid is unable to produce infectious chimeric virions. Not all regions of the calicivirus genome are likely to incorporate exogenous sequences. The insertion of the GFP gene at the start of ORF1 of TV completely abolished the infectivity of the transcribed RNA, which was not capable of expressing the fluorescent protein either [93]. This is why, in recent years, studies have been conducted to identify regions that tolerate insertions [102] and allow greater effectiveness in obtaining labeled reporter replicons [90]. The case of NV replicons requires special mention since a cell line stably expressing the replicon has been established [106]. For this, a fragment of the coding region of the VP1 gene was replaced by the gene for neomycin resistance, providing the mechanism for selecting cells that have incorporated the replicon. This system has further allowed studies on the replication of this non-cultivable virus [107,108] and the evaluation of specific inhibitors [109,110].

During the COVID-19 pandemic, unveiling SARS-CoV-2 behavior became crucial. Thus, a versatile reverse genetics platform for this virus was developed based on the circular polymerase extension reaction (CPER) methodology [111,112]. By generating overlapping cDNA fragments from viral RNA and assembling them along with a linker fragment containing a CMV promoter, a circular full-length viral cDNA is formed in a single reaction. Upon transfection of this circular cDNA into mammalian cells, an infectious SARS-CoV-2 virus was recovered. This system has been extended to *Alphamesonivirus 4* (Casuarina virus), *Ross River virus*, and human and murine noroviruses [22]. CPER for MNV-1 was developed by amplifying fragments from the plasmid DNA pSPORT-T7-MNV1, harboring the full-length MNV cDNA clone under the control of the T7 promoter. Three fragments, covering the complete MNV genome and featuring 27–34 nucleotide overlaps, were PCR-amplified. These amplicons were subsequently assembled into full-length cDNA and circularized with a linker fragment containing a 30-mer poly-A tail. In the case of human norovirus, total RNA was extracted from a clinical sample and cDNA was synthesized using an oligo-dT primer. This cDNA was then used to amplify three fragments that span the complete viral genome, and the final construct was circularized as for MNV-1. For both infectious clones, replication-competent viruses were rescued upon transfection of NIH3T3 cells [22].

A reverse genetics system was obtained for an infectious human sapovirus (HuSaV). The full-length RNAs from the HuSaV GII.3 and AK11 strains, both capped and uncapped, were produced through in vitro transcription and used to transfect HuTu80 (human duodenum carcinoma) cells. Infectious virions were successfully recovered from the cells transfected with capped RNA, confirming the relevance of the 5′-cap structure for virus recovery [113].

Another special mention deserves the alleged establishment of a reverse genetics system and cultivation method for rabbit hemorrhagic disease virus (RHDV) (genus *Lagovirus*), which, like human noroviruses and sapoviruses, has been refractory to propagation in cell cultures for many years. This work, among other extraordinary results, demonstrated that the synthetic genome of RHDV obtained through in vitro transcription of a cDNA clone with the SP6 phage polymerase and devoid of any protective RNA structure and translation enhancer (such as VPg or cap), is infectious and triggers hemorrhagic disease when inoculated into rabbits [114]. Furthermore, the authors suggest that viruses recovered from transfected rabbits adapted to cultivation in the RK13 cell line (derived from rabbit kidney). This culture-adapted virus was subsequently cloned into a vector under the control of a CMV promoter, which was capable of generating viral genomic transcripts by pol II action in the nucleus, ultimately giving rise to the progeny of infectious viruses [115]. These authors assert that surprisingly, neither the deletion of the VP2 coding region nor the absence of the poly-A tail prevents virus rescue, and furthermore, the poly-A tail is restored during replication [115,116]. The entirety of these results stands in stark contrast to what has been found to date for most caliciviruses, and unfortunately, none of these data have been validated by publications from other research groups experienced in working with this pathogen.

### The Rabbit Vesivirus Reverse Genetics Journey

Rabbit vesivirus (RaV) was first isolated from domestic rabbits’ feces in Portland (Oregon, USA) and subsequently identified and characterized in our laboratory [44,61,117]. Since then, the development of a reverse genetics system for RaV has been a primary goal in our research group for many years. Multiple obstacles had to be overcome in the pursuit of consistent infectious virus recovery. Most of the available reverse genetics strategies have been attempted, including the transfection of permissive cells with either synthetic genome-length RNA transcripts obtained in vitro or genome-encoding cDNA vectors ready for in vivo transcription, thereby minimizing RNA manipulation, potential exposure to contaminating RNases, and undesired degradation.

Viral progeny from RaV was successfully rescued once from a plasmid containing the RaV genome under the T7 phage RNA polymerase promoter and an *Xho*I restriction site (not found in the wild-type virus) as a molecular tag. This cDNA vector was named pTA23/Xh and, together with the superinfecting helper virus rFPV-T7, allowed the recovery of a progeny of RaV confirmed to harbor the *Xho*I molecular tag (unpublished data). Regrettably, despite this early successful RaV rescue, subsequent attempts failed to yield additional infectious viral particles from the same construct. Ensuing RaV genome-expressing vectors were derived from the genomic RNA extracted from the *Xho*I-tagged virus, rescued on that occasion (e.g., pT7-RaV and pT7-RaV/Xh).

Multiple RaV genome-encoding cDNA vectors were designed that gradually incorporated improvements aimed at producing viral genomic transcripts with an authentic 5′–end and a defined 3′–end, flanked only by the poly-A tail, which is naturally present in the *Caliciviridae*. These vectors carried different RNA polymerase promoters (SP6, T7, and CMV) and, in cases where the sequence of such promoters spanned beyond the +1 transcription site, truncated versions of the promoters were produced by removing the nucleotides following the +1 site, thus preventing the introduction of non-viral nucleotides at the 5′–end of the RNA. A ribozyme was placed at the 3′–end for the generation of authentic 3′–ends. Despite these improvements, no CPE was observed in passage 1 cultures in the very first attempts, suggesting the lack of a successful virus rescue. Since VP1 protein could not be detected in cell monolayers from passage 0 (transfected cells), it was assumed that the absence of capsid assembly was the cause of the failed rescues. Several auxiliary plasmids constitutively expressing each of the mature peptides derived from the ORF1-encoded wild-type RaV polyprotein, as well as wild-type VP1 and VP2, were separately or combinedly co-transfected with the full-length genome-expressing cDNA vector to address whether these gene products could somehow resume virus recovery when provided *in trans*. No virus recovery was achieved during these experiments, suggesting the occurrence of additional defects in the RaV infectious clone.

A meticulous sequence analysis of all RaV constructs in our laboratory revealed a single nucleotide change in all constructs that failed to generate infectious RaV. This nucleotide change consisted of an A-to-G transition within the 3′–UTR at position 8288, i.e., exactly eight nucleotides upstream from the first A of the poly-A tail. While all RaV infectious cDNA clones tested in our laboratory (including pTA23/Xh, pT7-RaV, and pT7-RaV/Xh) contain a G nucleotide, the original wild-type RaV contains an A at that position. More interestingly, the virus once rescued from the pTA23/Xh construct also contains an A, suggesting the occurrence of a unique reversion event that allowed such virus recovery just one time. When the correct nucleotide (A) was placed at position 8288 of the genome within the constructs pT7-RaV and pT7-RaV/Xh, those cDNA clones became infectious and the recovery of RaV from such clones became reproducible [43]. In addition to this correction, we also doubled the length of the poly-A tail of RaV infectious clones, which ultimately increased rescued virus yields likely due to increased viral RNA stability. This achievement was accomplished by transfecting permissive cells with plasmids that encode the full genome-length cDNA, driven by the T7 phage RNA polymerase. This enzyme was provided in trans by infecting the cells with rFPV-T7 prior to transfection. Likewise, infectious RaV was also successfully recovered when the transcription step was conducted in vitro followed by synthetic genome-length RNA transfection, as long as a 5′–cap structure was added to the 5′–end of the synthetic genome-length RNAs, either co- or post-transcriptionally [43].

**Table 2:** Calicivirus reverse genetics systems. The calicivirus infectious clones are listed chronologically, and the fundamentals of each reverse genetics strategy followed are briefly explained. The third column shows the general pattern observed for each infectious clone’s construction, according to the generalization depicted in Figure 6. Legend: IVT, in vitro transcription; rMVA-T7, Ankara-modified recombinant vaccinia virus expressing T7 RNA polymerase; rVV-T7, recombinant vaccinia virus expressing T7 RNA polymerase; rFPV-T7, recombinant fowlpox virus expressing T7 RNA polymerase; minCMV, minimal cytomegalovirus promoter. See the text for further details.

Virus	Recovery Strategy and Infectious Clone Features	Design	Year of Publication [Reference]
Feline calicivirus	T7 RNA polymerase-driven IVT with co-transcriptional capping, followed by RNA transfection of CRFK cells	I	**1995** [2]
Feline calicivirus	T7 RNA polymerase-driven cDNA expression; Poly-(A)_32_. Two delivery methods: transfection of CRFK cells with IVT-derived RNA (co-transcriptional capping) cDNA transfection of cells infected with rMVA-T7	I	**2002** [92]
Porcine enteric calicivirus	T7 RNA polymerase-driven cDNA expression; Poly-(A)_35_. Two delivery methods: transfection of LLC-MK2 cells with IVT-derived RNA (co-transcriptional capping) cDNA transfection of rMVA-T7-infected cells	I	**2005** [76]
Human norovirus	T7 RNA polymerase promoter: transfection of rMVA-T7-infected 293T cells; Poly-(A)_26_	III	**2005** [78]
Human norovirus	T7 RNA polymerase promoter: transfection of rVV-T7-infected 293T cells; Poly-(A)_30_	III	**2006** [77]
Human norovirus	No virus rescue: neomycin-resistance gene replacing part of ORF2. Transfection of BHK21 and Huh7 cells with IVT-generated RNA led to the establishment of a VP1-defective replicon that persisted beyond cell passages. Apparently, the replicon further extracted from cells had covalently acquired the 5′–linked VPg. G418 was used for colony selection	IV	**2006** [106]
Murine norovirus–1	Pol-II-driven: viral cDNA controlled by minCMV promoter; Poly-(A)_31_. Two delivery methods: transduction of HepG2 cells with an inducible baculovirus transfection of the cDNA into 293T cells	II	**2007** [95]
Murine norovirus–1	T7 RNA polymerase-driven cDNA expression; Poly-(A)_26_. Two helper viruses tested for providing T7 pol: rMVA-T7 (showed deleterious effect over MNV rescue) rFPV-T7 (allowed virus rescue)	II	**2007** [73]
Tulane virus	T7 RNA polymerase-driven IVT with co-transcriptional capping, followed by RNA transfection of LLC-MK2 cells; Poly-(A)_17_	I	**2008** [93]
Murine norovirus–1	T7 RNA polymerase-driven IVT. Post-transcriptional capping is used for the first time; RNA is delivered into RAW264.7 cells through electroporation T7 RNA polymerase-driven cDNA expression in BSR-T7 cells (constitutively expressing T7-pol); Poly-(A)_26_	II	**2010, 2012** [32,118]
Human norovirus	Pol-II-driven cDNA expression: EF-1α promoter. cDNA plasmid was transfected into COS7 cells in the absence of helper virus; Poly-(A)_26_	II	**2014** [105]
Feline calicivirus	Pol-II-driven cDNA expression: EF-1α promoter. cDNA plasmid was transfected into CRFK cells in the absence of helper virus; Poly-(A)_30_	II	**2014** [96]
Human norovirus	Pol-II-driven cDNA expression: CMV promoter. cDNA plasmid constructed through Gibson assembly and transfected into Caco-2 cells; bovine growth hormone; poly-A signal	I	**2018** [97]
Rabbit vesivirus	T7 RNA polymerase-driven cDNA expression; Poly-(A)_30_. Two delivery methods: transfection of 293T cells with IVT-derived RNA (post-transcriptional capping) cDNA transfection of rFPV-T7-infected 293T cells	III	**2020** [43]
Human norovirus Murine norovirus–1	Full-length cDNA with a linker fragment containing CMV promoter synthesized by CPER; transfected in NIH3T3 cells; Poly-(A)_30_	II	**2021** [22]
Human sapovirus	T7 RNA polymerase-driven IVT with co-transcriptional capping, followed by RNA transfection of HuTu80 cells; Poly-(A)_25_	I	**2022** [113]

## 6. Concluding Remarks and Future Perspectives

The recovery of infectious virions from genomic cDNA is not a routine procedure in the laboratory. Unfortunately, there are no universal protocols or fixed rules applicable to all caliciviruses. The systematic failure in the establishment of reverse genetics for difficult-to-cultivate caliciviruses such as RHDV, for which a reliable infectious clone is still lacking, is proof of this argument. Moreover, the diversity of reverse genetics strategies attempted by researchers and the intensive use of genetic engineering techniques to modify promoters, achieve precise transcription termination, or promote proper transcript processing exemplify the complexity of this methodology.

The techniques and workflow summarized in this review, based on the replication cycle of caliciviruses, have contributed to addressing which replication events proceed normally and which ones do not, bringing us closer to identifying the putative reasons behind failures of calicivirus rescue and establishing guidelines for correcting flaws in prospective experiments. Many of the insights in this review stem from our laboratory experience in constructing full-length viral cDNA clones using various strategies. While some perspectives may be subjective, this straightforward depiction aims to assist researchers unfamiliar with the calicivirus field in selecting the most suitable reverse genetics system for their studies and analyzing its performance in a step-by-step fashion.

Reverse genetics systems developed for members of the *Caliciviridae* family have played a pivotal role in studying such non-cultivable pathogens. While many significant questions remain unanswered, the increasing availability of infectious clones has significantly bolstered our capacity to address these questions.

Due to the obligate intracellular parasitic nature of viruses, virus reverse genetics experiments have traditionally relied on cell cultures, although entire host organisms have also been employed to a lesser extent. In recent years, we have witnessed a remarkable transformation in virus research, with the use of cultured organoids as a host model system for such experiments. The recent development of human intestinal enteroids (HIE) supported the replication of human sapoviruses and noroviruses, two caliciviruses among the most refractory to in vitro propagation [119,120]. This organoid model also provided a platform for the screening of compounds with potential antiviral effects that ultimately led to the discovery of dasabuvir, a human norovirus replication inhibitor [121]. Though a comprehensive discussion on the relevance of this novel model for virus reverse genetics is out of the scope of this review, we would like to emphasize its strong potential as the field quickly moves from classic cell culture to organoids. This revolutionary technique may allow the generation of an infectious RHDV clone by using hepatobiliary organoids derived from rabbits and other lagomorphs [122]. As this field is continuously growing, it is worth mentioning the importance of tackling any biosafety issue and setting up appropriate regulatory measures that guarantee the safe manipulation and containment of recombinant viruses.

In summary, the calicivirus reverse genetics research stands on the brink of exciting findings and breakthroughs. The modification of caliciviral genomes brings new opportunities for precise antiviral therapies targeting specific viral genes, the establishment of the molecular basis of virus attenuation, and the development of novel vaccine strategies. It is expected that the methodologies discussed in this review, in combination with other powerful technologies such as high-throughput screening, CRISPR/Cas gene editing, OMICS, and next-generation sequencing techniques, as well as the extensive implementation of cultured organoids, will lead us to safer and more efficacious vaccines and antivirals against caliciviruses and also to a better understanding of these pathogens that emerged long ago and have coexisted with humans and animals ever since.

## Figures and Tables

**Table 1:** Analyzing the occurrence of critical events in the viral replication cycle.

Promoter	Bacteriophage (T3, T7, SP6, etc.)			Eukaryotic (CMV, EF-1α, SV40, etc.)
**Reverse genetics strategy**	Transfection of in vitro-transcribed RNA	cDNA transfection of helper virus-infected cells	cDNA transfection of phage RNA pol-expressing cell line (no helper virus)	RNA pol II-driven nuclear transcription of cDNA
**Is there virus-induced CPE in transfected cell monolayers (passage 0)?**	Microscopic examination of transfected cells			
**Does the supernatant from transfected cells (passage 0) contain infectious virions?**	Blind passage of the supernatant from transfected cells and microscopic examination of inoculated (passage 1) monolayers			
**Is the genome-emulating RNA transcript present in the cytosol?**	Extraction of total cell RNA followed by detection of viral RNA through RT-PCR; emphasis should be given to RNA integrity (intactness and overall quality; lack of RNA degradation)			
**Is there any non-viral sequence inadvertently added to the 5′ or 3′–end?**	5′–RACE/3′–RACE assays			
**Is ORF1 being expressed?**	Western blot (WB) or Immunofluorescence (IF) using antibodies specific to viral proteins			
**Is the viral protease functional?**	WB, focusing on the expected sizes of ORF1-derived mature peptides Radioactive labeling and autoradiography			
**Is the viral RdRp functional?**	5BR assay			
**Is the negative strand being synthesized?**	Northern blot RT-PCR with primers specific for negative strand			
**Are VP1 or VP2 being synthesized?**	WB with specific antibodies for the detection of VP1 or VP2			

## Data Availability

Not applicable.
